# Transoral thyroidectomy in a consecutive cancer-enriched cohort of 1–000 patients: a single-surgeon experience with endoscopic and robotic platforms

**DOI:** 10.3389/fendo.2026.1877246

**Published:** 2026-09-30

**Authors:** Nam Kyung Kim, Junu Kim, Jun Sung Lee, Hyeok Jun Yun, Seok-Mo Kim, Yong Sang Lee, Hang-Seok Chang

**Affiliations:** Department of Surgery, Thyroid Cancer Center, Gangnam Severance Hospital, Institute of Refractory Thyroid Cancer, Yonsei University College of Medicine, Seoul, Republic of Korea

**Keywords:** endoscopy, oral surgical procedures, robotic surgical procedures, thyroid neoplasms, thyroidectomy

## Abstract

**Introduction:**

Transoral vestibular thyroidectomy, performed using an endoscopic (transoral endoscopic thyroidectomy via the vestibular approach [TOETVA]) or robot-assisted (transoral robotic thyroidectomy [TORT]) approach, is a scar-concealing alternative to open thyroidectomy. Given the paucity of large-scale single-surgeon studies spanning both platforms, the aim of this study was to characterize the clinicopathological and perioperative outcomes of a large cancer-enriched transoral thyroidectomy series and to examine differences between TOETVA and TORT.

**Methods:**

We retrospectively reviewed 1–000 consecutive patients who underwent TOETVA (n=450) or TORT (n=550) performed by a single endocrine surgeon between September 2016 and May 2024. An additional propensity score-matched analysis incorporating clinical characteristics and calendar time, with exact matching on extent of surgery, was performed in the contemporaneous cohort after TORT introduction, yielding 221 matched pairs.

**Results:**

Among the 1–000 cases, 986 (98.6%) were malignant, predominantly papillary thyroid carcinoma, with 99.8% at pathological stage I. Overall perioperative outcomes were acceptable, with low complication rates and infrequent drain use; recurrent laryngeal nerve palsy and mental nerve injury were confined to the early TOETVA period. In the overall cohort, TORT patients had larger tumors and more frequent T1b and N1a disease, with shorter operative time and hospital stay. After propensity score matching, between-platform differences were modest: TORT was associated with a 5.05-min longer operative time (95% CI, 0.39–9.72) and a 0.79-node higher central compartment node yield (95% CI, 0.18–1.41), whereas histopathological characteristics, hospital stay, and measured postoperative complications did not differ significantly. Sensitivity analyses yielded similar operative-time estimates.

**Conclusions:**

In this large cancer-enriched single-surgeon cohort, transoral thyroidectomy was associated with acceptable perioperative outcomes in carefully selected patients. Although TOETVA and TORT showed differences in the overall cohort, adjusted differences were modest and did not establish superiority of either platform. Both approaches may retain practical value within an evolving transoral thyroidectomy practice.

## Introduction

Conventional open thyroidectomy (COT) has traditionally been considered the reference standard for safety and oncologic adequacy (1, 2). However, visible anterior neck scars and the potential for postoperative pigmentary changes remain major concerns for many patients owing to their effects on the quality of life (2, 3). Therefore, remote-access and minimally invasive techniques have been developed to preserve surgical quality while reducing or eliminating a cervical skin incision (1, 4).

Among such methods, the transoral vestibular approach is a distinctive option because it avoids a cutaneous neck incision while preserving direct midline access to the thyroid gland and central compartment (5, 6). This approach can be performed using two principal techniques: a purely endoscopic method, known as transoral endoscopic thyroidectomy via the vestibular approach (TOETVA), and a robot-assisted method, referred to as transoral robotic thyroidectomy (TORT) (5, 7).

Both TOETVA and TORT are feasible and have been reported to achieve outcomes comparable to those of COT, with potential advantages in selected domains (4, 8, 9). Nevertheless, most of the available literature is limited by heterogeneous patient populations, relatively small numbers of malignant cases, or a focus on a single surgical platform. In addition, comprehensive studies from high-volume single-surgeon practices spanning both platforms, particularly in cancer-enriched cohorts, remain limited (6, 7, 10).

To address this gap, we analyzed a consecutive series of 1–000 transoral thyroidectomies performed by a single surgeon. This cohort, which included 986 malignant cases (98.6%), is one of the largest single-surgeon transoral thyroidectomy series enriched for malignant disease and encompassing both endoscopic and robot-assisted approaches.

The aim of this study was to characterize the overall clinicopathological features and perioperative outcomes of this large cancer-enriched series and to examine differences between TOETVA and TORT within an evolving clinical practice environment.

## Materials and methods

### Ethical considerations

This study was conducted in accordance with the Declaration of Helsinki (as revised in 2013) and was approved by the Institutional Review Board of Gangnam Severance Hospital, Yonsei University College of Medicine (Seoul, Korea) (approval number: 3-2025-0306). The requirement for individual informed consent was waived because of the retrospective nature of this analysis.

### Study design and patient selection

This single-center retrospective cohort study was reported in accordance with the STROCSS 2025 guideline and the STROBE statement (11, 12). The study was not preregistered, as it was a retrospective observational analysis of routinely collected clinical data. The medical records of 1–000 consecutive patients who underwent transoral vestibular thyroidectomy, via either TOETVA or TORT, performed by a single endocrine surgeon between September 2016 and May 2024 were retrospectively reviewed.

Patients with cytologically or radiologically suspected thyroid cancer or benign thyroid disease were eligible for inclusion. Indeterminate nodules with Bethesda III–IV cytology on fine-needle aspiration were included when surgery was recommended for interval growth, compressive symptoms, or patient preference.

Patients were excluded if imaging or clinical factors were incompatible with safe or effective transoral access. Exclusion criteria included gross extrathyroidal extension (ETE) with suspected invasion of adjacent organs on ultrasonography (US) or contrast-enhanced computed tomography (CT); bulky central or lateral lymph node metastases requiring extended compartment dissection; a history of major anterior-neck surgery or irradiation with markedly distorted planes; active oral infection or uncorrected poor dental hygiene; and other anesthetic or surgical contraindications to subplatysmal carbon dioxide (CO_2_) insufflation.

TOETVA was introduced in September 2016, whereas TORT was introduced in March 2020. Before TORT became available, all transoral procedures were performed using TOETVA. Thereafter, selection of the surgical technique (TOETVA vs. TORT) was not randomized but was determined through shared decision-making. This process integrated disease-related factors (nodule size and location, cytology, and US/CT risk features), patient priorities (cosmetic outcomes and cost considerations), and logistical factors (platform availability and operating room scheduling).

### Surgical procedures

The procedure’s details have been well-documented in prior publications from our center (13). Briefly, TOETVA and TORT were performed using a standardized three-port transoral vestibular approach with low-pressure CO_2_ insufflation. Following flap dissection, the strap muscles were divided via the midline and retracted using a purpose-built endoscopic thyroid retractor to maintain exposure without additional ports (**Figure 1**).

**Figure 1:**
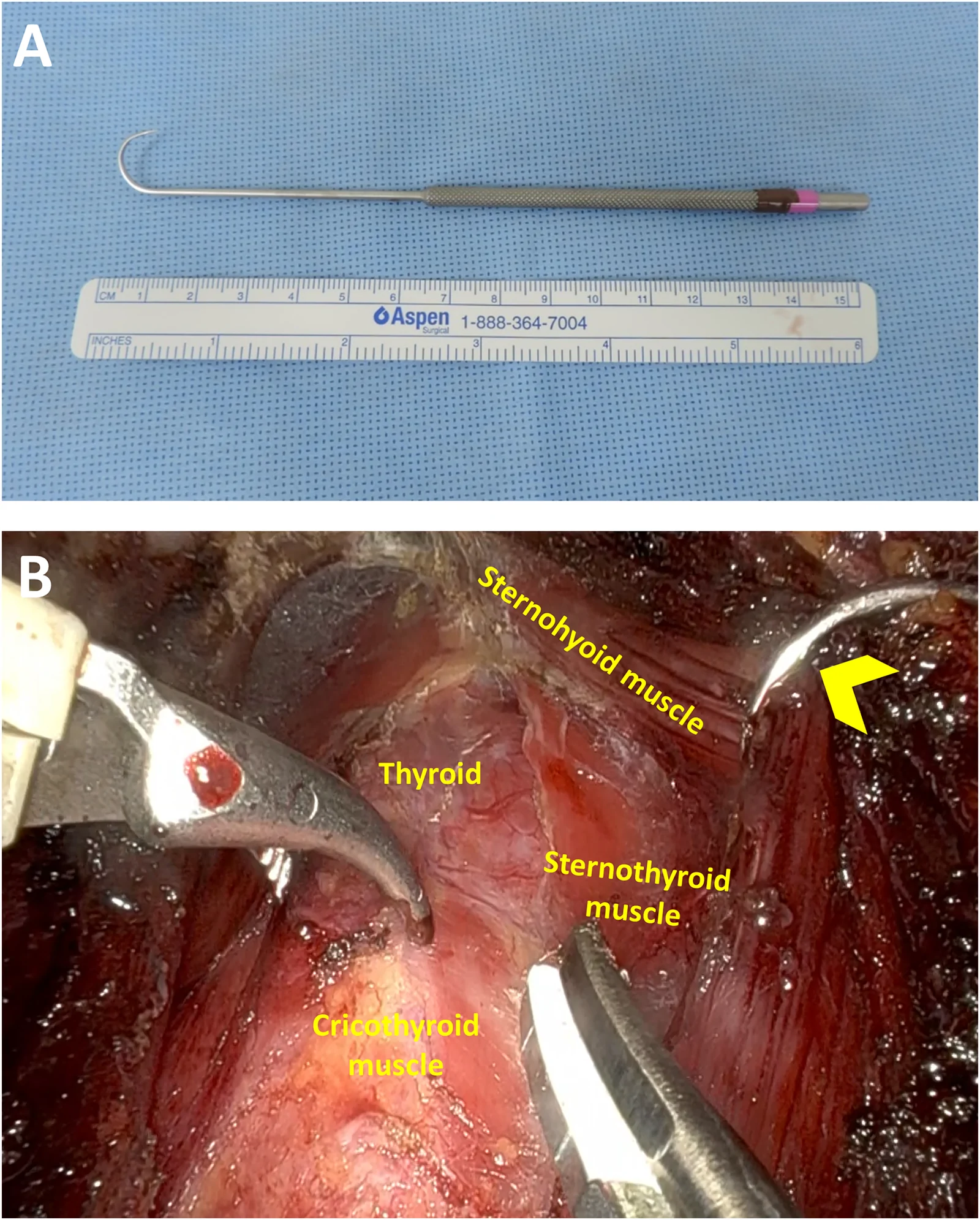
Purpose-built retractor used in transoral thyroidectomy. **(A)** Design of the retractor. **(B)** Intraoperative view illustrating the retractor (yellow arrowhead) positioned around the strap muscles and repositioned between the superficial and deep strap layers to maintain the working space without additional external ports.

Next, thyroidectomy was performed with identification and preservation of the recurrent laryngeal nerve (RLN) and parathyroid glands. In this series, most cases involved nodules with high clinical or radiologic suspicion of malignancy; therefore, ipsilateral prophylactic central compartment neck dissection (CCND) was incorporated into the institutional transoral workflow at our center.

For TORT, vestibular access and flap creation were identical to those used in TOETVA before docking of the da Vinci Xi robotic system. Time intervals were defined as follows: docking time was measured from the completion of vestibular port placement to completion of robot docking, and console time was measured from the start of robotic dissection to completion of thyroid resection and hemostasis before undocking.

**Figures 2A, C, E** show representative superior-pole dissection, RLN identification, and CCND for TOETVA, and **Figures 2B, D, F** show those for TORT.

**Figure 2:**
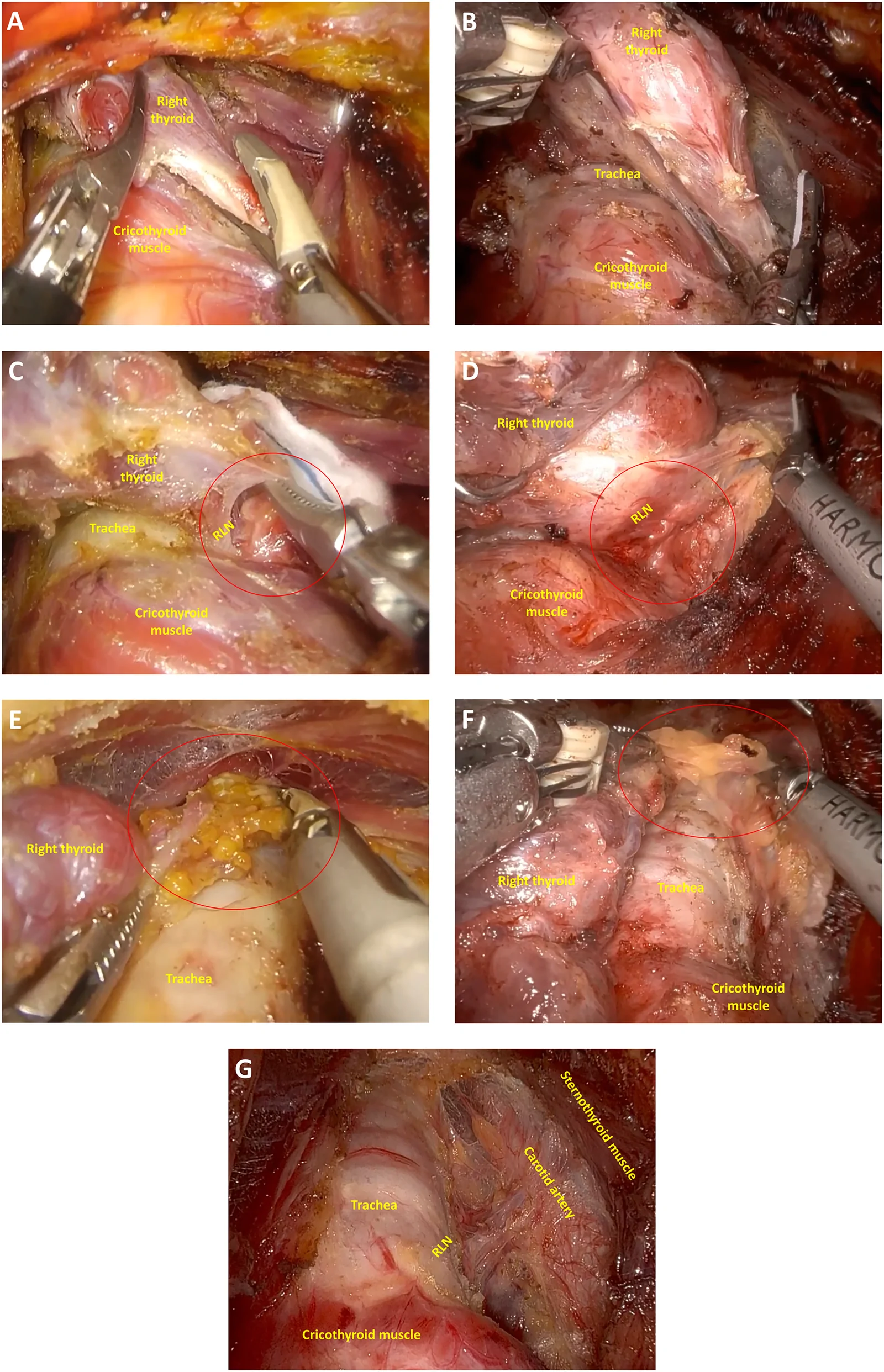
Intraoperative views of key surgical steps in TOETVA and TORT. **(A)** Superior pole dissection and ligation of the superior thyroid vessels during TOETVA. **(B)** Corresponding superior pole dissection during TORT using the robotic platform. **(C)** Identification of the RLN at its laryngeal entry point during TOETVA. **(D)** Identification of the RLN during TORT. **(E)** Ipsilateral prophylactic CCND during TOETVA. **(F)** Ipsilateral prophylactic CCND during TORT. **(G)** Final operative view following thyroidectomy demonstrating major central neck anatomical landmarks. TOETVA, transoral endoscopic thyroidectomy via the vestibular approach; TORT, transoral robotic thyroidectomy; RLN, recurrent laryngeal nerve; CCND, central compartment neck dissection.

Surgical drains were generally not used for either TOETVA or TORT, except under special circumstances.

### Postoperative management and follow-up

A single additional dose of intravenous antibiotic was administered postoperatively. After recovery from general anesthesia, patients were allowed to drink water, and a soft diet was initiated 6 h post-surgery. Most patients were discharged within 1–3 days postoperatively. Patients were followed postoperatively in the outpatient clinic in accordance with routine institutional practice. Adjuvant radioactive iodine (RAI) therapy was administered after total thyroidectomy when indicated based on pathological risk factors.

### Outcome measurement

Clinicopathological variables, operative details, and postoperative outcomes were obtained from operative video review and electronic medical records. Tumor staging was determined according to the 8th edition of the American Joint Committee on Cancer (AJCC) TNM staging system (14).

Hypoparathyroidism was defined as a parathyroid hormone (PTH) level <15 pg/mL at 24 h postoperatively or symptomatic hypocalcemia requiring calcium supplementation (1, 3, 5). RLN injury was defined as new-onset hoarseness and/or impaired motion of one or both vocal cords on postoperative laryngoscopy when clinically indicated (5). Mental nerve function was assessed during hospitalization and at the outpatient follow-up by asking patients about paresthesia or numbness in the chin or lower lip (15). A 6-month cutoff was applied to differentiate transient from permanent complications. Permanent hypoparathyroidism was defined as an ongoing requirement for calcium or active vitamin D supplementation or a serum PTH level <15 pg/mL at 6 months. Oral wound infection was defined as a surgical-site infection requiring additional antibiotic therapy or an incision-and-drainage procedure (7). Seroma was documented during needle aspiration. Bleeding was recorded when reoperation for hemostasis was necessary. Chyle leakage was defined as postoperative lymphatic leakage requiring conservative management (e.g., nil per os or local gauze tamponade) or reoperation for lymphatic ligation.

### Statistical analysis

Continuous variables were summarized as mean ± standard deviation or median (interquartile range), as appropriate, and categorical variables as number (percentage). Unadjusted comparisons were performed using the chi-square or Fisher’s exact test for categorical variables and the independent-samples t test or Mann–Whitney U test for continuous variables, as appropriate. Because platform selection was not randomized, comparisons in the overall cohort were considered descriptive.

An additional propensity score-matched analysis was performed in the contemporaneous period after TORT introduction to reduce differences in case mix and operative era. Propensity scores were estimated using sex, age, BMI, nodule size, thyroiditis, and months since TORT introduction. One-to-one nearest-neighbor matching without replacement was performed, with exact matching on extent of surgery. Covariate balance was assessed using absolute standardized mean differences, with values <0.10 considered acceptable. Matching yielded 221 pairs.

Matched outcomes were analyzed using generalized estimating equations with robust standard errors to account for matched-pair clustering. Mean differences were estimated for continuous outcomes and risk differences for categorical outcomes, with effect estimates expressed as TORT minus TOETVA. Histopathological comparisons were restricted to 212 matched pairs in which both patients had malignant disease. Sensitivity analyses for operative time additionally accounted for calendar time or cumulative transoral experience in separate models because of substantial collinearity. Because TOETVA and TORT share vestibular access, flap creation, and a craniocaudal operative view, cumulative transoral experience was considered potentially relevant to operative performance. However, the robotic thyroidectomy phase requires platform-specific adaptation; therefore, robot-specific experience was evaluated separately using the number of prior TORT cases. Exploratory restricted cubic spline analyses were used to evaluate associations between the number of prior TORT cases and total operative, docking, and console times.

A two-sided p value <0.05 was considered statistically significant. Statistical analyses were performed using SAS version 9.4 (SAS Institute, Cary, NC, USA) and R version 4.3.2 (R Foundation for Statistical Computing, Vienna, Austria). Propensity score matching was performed using the MatchIt package in R.

## Results

### Baseline characteristics of the overall cohort (N = 1 000)

A total of 1–000 consecutive transoral thyroidectomies performed by a single surgeon were analyzed, comprising 450 TOETVA and 550 TORT cases. The cohort was predominantly female (n = 913; 91.3%), with a mean age of 37.06 ± 8.21 years and a body-mass index (BMI) of 22.92 ± 3.71 kg/m². A BMI ≥25 kg/m² was observed in 230 patients (23.0%).

Lobectomy was the most common procedure (n = 823; 82.3%); the mean diameter of the index nodule was 0.81 ± 0.46 cm. Overall, 986 patients (98.6%) had malignant disease, predominantly papillary thyroid carcinoma (PTC) (n = 977; 97.7%). Concomitant lymphocytic thyroiditis was present in 337 patients (33.7%). The mean number of retrieved central compartment lymph nodes was 3.32 ± 3.16 (**Table 1**).

**Table 1:** Baseline characteristics of the overall cohort and according to the surgical platform.

Variable	Overall (N = 1 000)	TOETVA (n = 450)	TORT (n = 550)	p-value
Sex (female), n (%)	913 (91.3)	417 (92.7)	496 (90.2)	0.165
Age (years), mean ± SD	37.06 ± 8.21	36.58 ± 8.09	37.45 ± 8.29	0.093
BMI (kg/m^2^), mean ± SD	22.92 ± 3.71	22.79 ± 3.55	23.03 ± 3.83	0.308
BMI ≥25, n (%)	230 (23.0)	104 (23.1)	126 (22.9)	0.940
Extent of surgery, n (%)				0.571
Total thyroidectomy with CCND	71 (7.1)	29 (6.4)	42 (7.6)	
Lobectomy with CCND	823 (82.3)	374 (83.1)	449 (81.6)	
Isthmusectomy with CCND	102 (10.2)	47 (10.4)	55 (10.0)	
Nodulectomy with CCND	2 (0.2)	0 (0.0)	2 (0.4)	
Lobectomy and nodulectomy with CCND	2 (0.2)	0 (0.0)	2 (0.4)	
Nodule size in the greatest diameter (cm), Mean ± SD	0.81 ± 0.46	0.72 ± 0.39	0.88 ± 0.51	<0.001
Main site, n (%)				0.962
Right	471 (47.1)	210 (46.7)	261 (47.5)	
Left	404 (40.4)	185 (41.1)	219 (39.8)	
Isthmus	124 (12.4)	55 (12.2)	69 (12.5)	
Final diagnosis, n (%)				0.354
PTC	977 (97.7)	442 (98.2)	535 (97.3)	
FTC†	5 (0.5)	2 (0.4)	3 (0.5)	
MTC	2 (0.2)	0 (0.0)	2 (0.4)	
OTC	2 (0.2)	2 (0.4)	0 (0.0)	
NIFTP	1 (0.1)	0 (0.0)	1 (0.2)	
Benign	13 (1.3)	4 (0.9)	9 (1.6)	
Thyroiditis, n (%)	337 (33.7)	149 (33.1)	188 (34.2)	0.722
Retrieved central compartment nodes, mean ± SD (range)	3.32 ± 3.16 (0–21)	2.87 ± 2.84 (0–21)	3.68 ± 3.35 (0–20)	<0.001

TOETVA, transoral endoscopic thyroidectomy via the vestibular approach; TORT, transoral robotic thyroidectomy; BMI, body mass index; CCND, central compartment neck dissection; PTC, papillary thyroid carcinoma; FTC, follicular thyroid carcinoma; MTC, medullary thyroid carcinoma; OTC, oncocytic thyroid carcinoma; NIFTP, noninvasive follicular thyroid neoplasm with papillary-like nuclear features; SD, standard deviation.

†All FTC cases were minimally invasive.

### Baseline comparison between TOETVA and TORT

In unadjusted descriptive comparisons, no significant differences were observed between the groups in terms of sex, age, BMI, operative extent, lesion laterality, and the prevalence of lymphocytic thyroiditis. The mean diameter of the index nodule was larger in the TORT group than in the TOETVA group (0.88 ± 0.51 vs. 0.72 ± 0.39 cm, p <0.001). The yield of central compartment lymph nodes was also higher with TORT than with TOETVA (3.68 ± 3.35 vs. 2.87 ± 2.84, p <0.001). Final diagnosis distributions were similar between the groups (p = 0.354), with PTC comprising most cases in both groups (98.2% in TOETVA vs. 97.3% in TORT) (**Table 1**).

### Cancer histopathology in the overall malignant cohort

Among the 986 cases in the overall malignant cohort, gross ETE was identified in 16 patients (1.6%). Lymphatic invasion was present in 240 patients (24.3%), and vascular invasion was observed in 155 (15.7%).

Pathologic staging was predominantly early, with stage I disease accounting for 984 cases (99.8%). T1 tumors predominated (96.4%), comprising 765 T1a lesions (77.6%) and 185 T1b lesions (18.8%). Pathologic N1a disease was documented in 306 patients (31.0%), and the *BRAF* mutation was observed in 792 (80.3%). The mean number of retrieved central compartment lymph nodes was 3.33 ± 3.16 (**Table 2**).

**Table 2:** Histopathological characteristics of malignant cases overall and according to the surgical platform.

Variable	Overall (N = 986)	TOETVA (n = 446)	TORT (n = 540)	p-value
Extrathyroidal extension, n (%)				<0.001
Intrathyroidal	916 (92.9)	395 (88.6)	521 (96.5)	
Minimal	54 (5.5)	42 (9.4)	12 (2.2)	
Gross	16 (1.6)	9 (2.0)	7 (1.3)	
Lymphatic invasion (present), n (%)	240 (24.3)	68 (15.2)	172 (31.9)	<0.001
Vascular invasion (present), n (%)	155 (15.7)	56 (12.6)	99 (18.3)	0.013
Retrieved central compartment nodes, mean ± SD (range)	3.33 ± 3.16	2.88 ± 2.85 (1–21)	3.70 ± 3.35 (1–20)	<0.001
Pathological TNM classification, n (%)				
T category				<0.001
T1a	765 (77.6)	373 (83.6)	392 (72.6)	
T1b	185 (18.8)	61 (13.7)	124 (23.0)	
T2	20 (2.0)	3 (0.7)	17 (3.1)	
T3b	16 (1.6)	9 (2.0)	7 (1.3)	
N category				<0.001
N0	680 (69.0)	332 (74.4)	348 (64.4)	
N1a	306 (31.0)	114 (25.6)	192 (35.6)	
Stage group				0.504
I	984 (99.8)	446 (100.0)	538 (99.6)	
II	2 (0.2)	0 (0.0)	2 (0.4)	
*BRAF* mutation status, n (%)				0.012
Positive	792 (80.3)	363 (81.4)	429 (79.4)	
Negative	165 (16.7)	58 (13.0)	107 (19.8)	
Not tested	29 (2.9)	25 (5.6)	4 (0.7)	

TOETVA, transoral endoscopic thyroidectomy via the vestibular approach; TORT, transoral robotic thyroidectomy; SD, standard deviation.

### Cancer histopathology by technique (TOETVA, n = 446; TORT, n = 540)

The overall distribution of ETE differed between the groups (p < 0.001), although gross ETE was uncommon in both TOETVA and TORT (2.0% vs. 1.3%). The TORT group had higher rates of lymphatic invasion (31.9% vs. 15.2%; p <0.001) and vascular invasion (18.3% vs. 12.6%; p = 0.013).

T1b tumors (23.0% vs. 13.7%, p <0.001) and N1a disease (35.6% vs. 25.6%, p <0.001) were also more frequent in the TORT group. The mean number of retrieved central compartment lymph nodes was higher in the TORT group (3.70 ± 3.35 vs. 2.88 ± 2.85, p <0.001). The distribution of *BRAF* mutation status differed between the groups (p = 0.012) (**Table 2**).

### Operative outcomes and complications in the overall cohort (N = 1 000)

Mean operative time for the overall cohort was 71.2 ± 25.6 min; 96.2 ± 28.9 min for total thyroidectomy, 70.2 ± 24.6 min for lobectomy, and 61.4 ± 21.3 min for isthmusectomy. A surgical drain was used in five patients (0.5%).

Complications were uncommon. Hypoparathyroidism occurred in 21 patients (2.1%), comprising 19 transient cases (1.9%) and 2 permanent cases (0.2%). Transient RLN palsy occurred in six patients (0.6%). Mental nerve injury occurred in five patients (0.5%; transient: four [0.4%], permanent: one [0.1%]). Oral wound infection was observed in three patients (0.3%), and seroma was observed in nine patients (0.9%). No cases of postoperative bleeding, chyle leakage, or skin flap burn were observed.

The length of hospital stay was 2.30 ± 0.69 days. RAI was administered to 18 of 71 patients (25.4%) who underwent total thyroidectomy, corresponding to 1.8% (18/1 000) of the entire cohort (**Table 3**).

**Table 3:** Operative outcomes and complications overall and according to the surgical platform.

Variable	Overall (N = 1 000)	TOETVA (n = 450)	TORT (n = 550)	p-value
**Total operative time**, mean ± SD (range)	71.2 ± 25.6 (28–295)	75.5 ± 28.0 (28–295)	67.7 ± 23.0 (34–202)	<0.001
Total thyroidectomy with CCND (n = 71)	96.2 ± 28.9 (44–202)	99.9 ± 28.3 (61–182) (n = 29)	93.6 ± 29.4 (44–202) (n = 42)	0.372
Lobectomy with CCND (n = 823)	70.2 ± 24.6 (28–295)	74.7 ± 27.3 (28–295) (n = 374)	66.5 ± 21.3 (34–202) (n = 449)	<0.001
Isthmusectomy with CCND (n = 102)	61.4 ± 21.3 (34–155)	66.3 ± 25.0 (34–155) (n = 47)	57.2 ± 16.6 (36–139) (n = 55)	0.030
Nodulectomy with CCND (n = 2)	77.0 ± 15.6 (66–88)		77.0 ± 15.6 (66–88) (n = 2)	
Lobectomy and nodulectomy with CCND (n = 2)	73.5 ± 6.4 (69–78)		73.5 ± 6.4 (69–78) (n = 2)	
Docking time, mean ± SD			3.1 ± 3.5	
Console time, mean ± SD			19.8 ± 12.9	
Surgical drain, n (%)	5 (0.5)	2 (0.4)	3 (0.5)	> 0.99
Complications, n (%)				
Hypoparathyroidism: transient/permanent*	19/2 (1.9/0.2)	5/1 (1.1/0.2)	14/1 (2.5/0.2)	0.174
Transient RLN injury	6 (0.6)§	6 (1.3)§	0 (0.0)	0.008
Mental nerve injury: transient/permanent	4/1 (0.4/0.1)||	4/1 (0.9/0.2)||	0/0 (0.0/0.0)	0.018
Oral wound infection	3 (0.3)	2 (0.4)	1 (0.2)	0.591
Seroma	9 (0.9)	5 (1.1)	4 (0.7)	0.739
Bleeding	0 (0.0)	0 (0.0)	0 (0.0)	
Chyle leakage	0 (0.0)	0 (0.0)	0 (0.0)	
Skin flap burn	0 (0.0)	0 (0.0)	0 (0.0)	
Length of hospital stay, days, mean ± SD (range)	2.3 ± 0.7 (1–11)	2.4 ± 0.8 (1–11)	2.2 ± 0.5 (1–6)	<0.001
Postoperative RAI, n/N (%)	18/71 (25.4)¶	7/29 (24.1)	11/42 (26.2)	0.845

TOETVA, transoral endoscopic thyroidectomy via the vestibular approach; TORT, transoral robotic thyroidectomy; RLN, recurrent laryngeal nerve; RAI, radioactive iodine therapy; CCND, central compartment neck dissection; SD, standard deviation.

*Permanent hypoparathyroidism occurred exclusively in the total thyroidectomy group (2/71, 2.82%).

§All six transient RLN palsy events occurred during the initial adoption phase in 2016.

||All cases of mental nerve injury were observed during the early phase of the series, with one permanent case in September 2016 and four transient cases between September 2016 and April 2017.

¶Denominator is limited to patients who underwent total thyroidectomy (n = 71); the proportion in the entire cohort is 1.8% (18/1 000). Bold values indicate statistical significance (p < 0.05).

### Operative outcomes and complications by technique

In the overall cohort, total operative time was shorter with TORT than with TOETVA (67.7 ± 23.0 vs. 75.5 ± 28.0 min, p <0.001). This difference was also observed for lobectomy and isthmusectomy, whereas operative time for total thyroidectomy did not differ significantly between techniques. For TORT, mean docking and console times were 3.1 ± 3.5 and 19.8 ± 12.9 min, respectively. Drain use was rare and comparable between the groups (0.5% vs. 0.4%, p > 0.99).

Complications were infrequent in both groups. Transient RLN palsy occurred in 1.3% (6/450) of TOETVA cases and 0% (0/550) of TORT cases (p = 0.008); all six transient RLN palsy events occurred during the earliest phase of the series in 2016. Mental nerve injury occurred only following TOETVA (transient: four [0.9%]; permanent: one (0.2%), p = 0.018), with all five events occurring during the early phase of the series in 2016–2017. Rates of hypoparathyroidism (transient and permanent), oral wound infection, and seroma did not differ significantly between techniques.

Mean length of hospital stay differed modestly, yet significantly, between groups (2.2 ± 0.5 days in TORT vs. 2.4 ± 0.8 days in TOETVA, p <0.001). Postoperative RAI therapy among patients who underwent total thyroidectomy did not differ between techniques (**Table 3**).

### Propensity score-matched analysis

After exclusion of 183 TOETVA procedures performed before TORT introduction, 817 procedures were eligible for propensity score matching (267 TOETVA and 550 TORT) (**Figure 3**). One-to-one matching yielded 221 matched pairs, with all post-match absolute standardized mean differences below 0.10 (**Supplementary Table S1**; **Supplementary Figure S1**).

**Figure 3:**
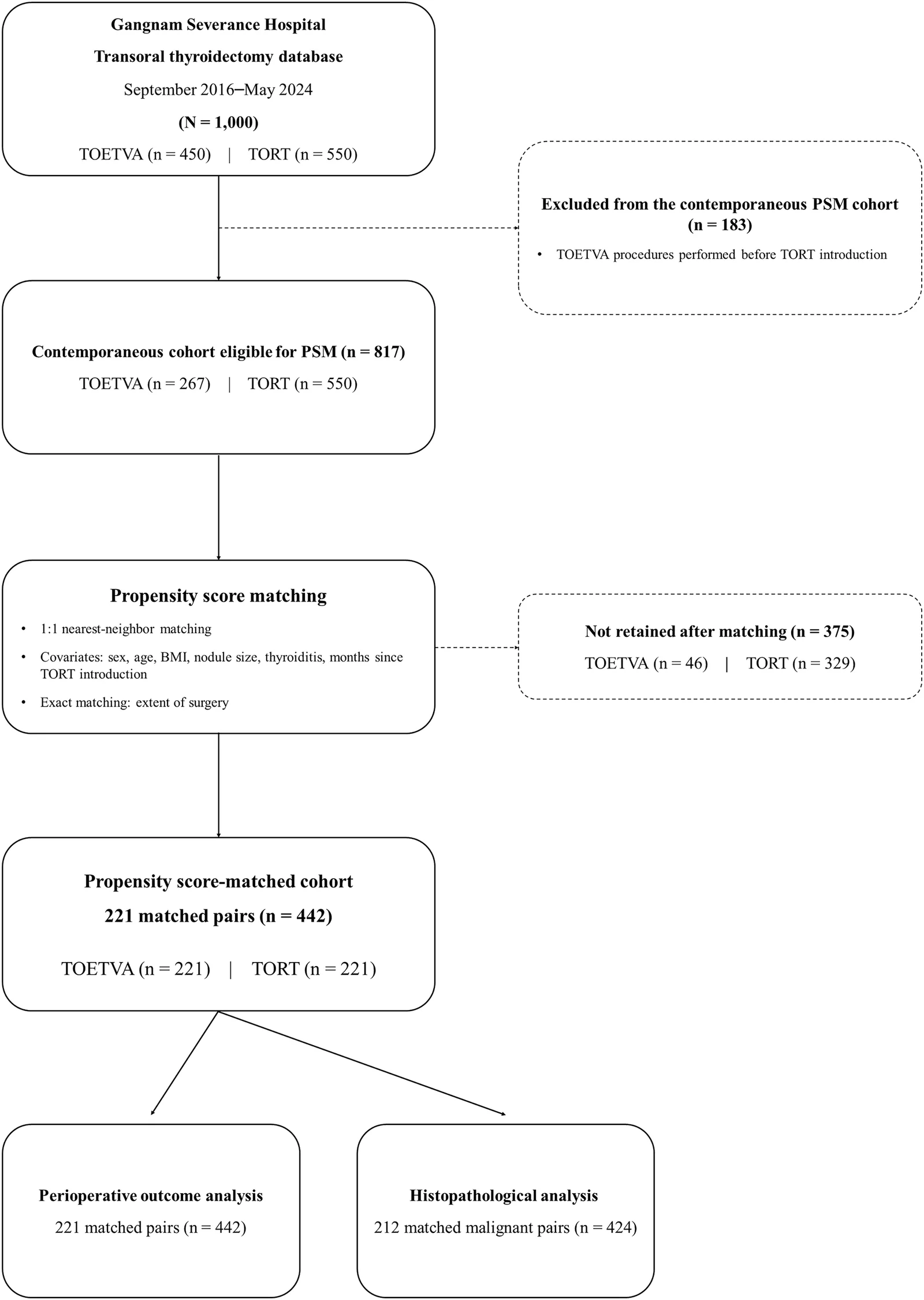
Study cohort and propensity score matching flow diagram. From 1–000 consecutive transoral thyroidectomy cases, 183 TOETVA procedures performed before TORT introduction were excluded from the contemporaneous matching cohort. One-to-one propensity score matching using sex, age, BMI, nodule size, thyroiditis, and months since TORT introduction, with exact matching on extent of surgery, yielded 221 matched pairs for perioperative outcome analysis. Histopathological analysis included 212 matched pairs in which both patients had malignant disease. PSM, propensity score matching; TOETVA, transoral endoscopic thyroidectomy via the vestibular approach; TORT, transoral robotic thyroidectomy; BMI, body mass index.

In the matched cohort, total operative time was modestly longer with TORT than with TOETVA (70.31 ± 26.42 vs. 65.26 ± 24.91 min; mean difference, 5.05 min; 95% CI, 0.39–9.72; p = 0.034). Central compartment node yield was also slightly higher with TORT (mean difference, 0.79 nodes; 95% CI, 0.18–1.41; p = 0.011), whereas hospital stay did not differ significantly (mean difference, −0.10 days; 95% CI, −0.20 to 0.01; p = 0.063). No significant differences were observed in hypoparathyroidism. No RLN injury, mental nerve injury, oral wound infection, or seroma occurred in either matched group (**Table 4**).

**Table 4:** Propensity score-matched comparison of perioperative outcomes between TOETVA and TORT.

Variable	TOETVA (n = 221)	TORT (n = 221)	Difference, TORT – TOETVA (95% CI)	p-value
Total operative time, min	65.26 ± 24.91	70.31 ± 26.42	5.05 (0.39 to 9.72)	0.034
Operative time by extent of surgery, min				
Total thyroidectomy with CCND	82.15 ± 16.95	85.31 ± 24.07	3.15 (−10.87 to 17.18)	0.659
Lobectomy with CCND	65.32 ± 25.34	70.68 ± 26.46	5.36 (0.01 to 10.72)	0.049
Isthmusectomy with CCND	54.24 ± 19.18	57.71 ± 22.53	3.48 (−4.52 to 11.48)	0.394
Retrieved central compartment nodes*	2.89 ± 3.02	3.68 ± 3.52	0.79 (0.18 to 1.41)	0.011
Surgical drain	0 (0.0)	0 (0.0)	—	—
Length of hospital stay, days	2.28 ± 0.70	2.18 ± 0.46	−0.10 (−0.20 to 0.01)	0.063
Complications				
Transient hypoparathyroidism†	1 (0.5)	4 (1.8)	1.4 (−0.2 to 2.9)	0.081
Permanent hypoparathyroidism†	0 (0.0)	1 (0.5)	0.5 (−0.4 to 1.3)	0.316
Transient RLN injury	0 (0.0)	0 (0.0)	—	—
Mental nerve injury	0 (0.0)	0 (0.0)	—	—
Oral wound infection	0 (0.0)	0 (0.0)	—	—
Seroma	0 (0.0)	0 (0.0)	—	—

Values are presented as mean ± SD, n (%), or n/N (%), as appropriate. Mean differences for continuous outcomes and risk differences for categorical outcomes, with 95% confidence intervals, were estimated using generalized estimating equations with robust standard errors to account for the matched-pair structure. Risk differences for categorical outcomes are expressed as absolute percentage-point differences. Effect estimates are expressed as TORT − TOETVA; positive values indicate higher or longer values in TORT.

*Retrieved central compartment node count was available for 219 patients in each group.

†All hypoparathyroidism events occurred among patients undergoing total thyroidectomy. Among the 13 matched total-thyroidectomy pairs, transient hypoparathyroidism occurred in 1/13 (7.7%) TOETVA and 4/13 (30.8%) TORT patients, whereas permanent hypoparathyroidism occurred in 0/13 (0.0%) and 1/13 (7.7%), respectively.

Effect estimates and P values were not calculated for outcomes with no events in either group.

TOETVA, transoral endoscopic thyroidectomy via the vestibular approach; TORT, transoral robotic thyroidectomy; CCND, central compartment neck dissection; RLN, recurrent laryngeal nerve; CI, confidence interval; SD, standard deviation.

Among the 212 malignant matched pairs, no significant between-platform differences were observed in gross ETE, lymphatic or vascular invasion, T category, pathological N1a disease, or BRAF mutation status (**Supplementary Table S2**).

### Sensitivity and exploratory learning-curve analyses

The estimated operative-time difference was similar after additional adjustment for calendar time (5.29 min; 95% CI, 0.62–9.95; p = 0.026) or cumulative transoral experience (5.14 min; 95% CI, 0.47–9.82; p = 0.031) (**Supplementary Table S3**). In exploratory analyses restricted to TORT procedures, cumulative robotic experience showed significant nonlinear associations with total operative, docking, and console times (**Supplementary Figure S2**).

## Discussion

In this consecutive single-surgeon series of 1–000 transoral thyroidectomies, 98.6% of patients had malignant disease, and perioperative outcomes were acceptable with low complication rates. Importantly, the apparent differences between TOETVA and TORT in the overall cohort were substantially altered after accounting for case mix and operative era. In the contemporaneous propensity score-matched cohort, TORT was associated with a modestly longer operative time and slightly higher central compartment node yield, whereas hospital stay, measured postoperative complications, and histopathological characteristics did not differ significantly. These findings emphasize that crude between-platform differences should not be interpreted as intrinsic advantages of either technique.

The scale and composition of this cohort also distinguish it from most previous transoral thyroidectomy series. Our series included 986 malignant cases (98.6%) among 1–000 consecutive transoral thyroidectomies. To our knowledge, this represents one of the largest single-surgeon datasets focused on transoral thyroidectomy enriched for malignant disease reported to date. Furthermore, both techniques were performed by the same surgeon within a single institutional practice using a standardized operative workflow, a key strength that minimizes inter-surgeon variability.

Major complications were uncommon, with no cases of postoperative bleeding or chyle leakage, infrequent drain use, and a short mean hospital stay. Approach-specific complications, such as mental nerve injury, occurred infrequently and were predominantly transient. The permanent hypoparathyroidism rate was 0.2%; however, when the analysis was restricted to the total thyroidectomy subgroup, the rate increased to 2.82% (2/71). This estimate should be interpreted with caution, as it is based on a relatively small denominator and may, therefore, be unstable. The higher subgroup-specific rate should also be considered in the context of the greater technical demands of parathyroid preservation during total thyroidectomy with central compartment dissection compared with less extensive surgery.

The observed difference in RLN palsy warrants cautious interpretation. Although transient RLN palsy occurred only in the TOETVA group, all six events occurred during the earliest phase of the series in 2016. Mental nerve injury showed a similar temporal pattern, with all five events occurring during 2016–2017. Moreover, neither RLN nor mental nerve injury occurred in either group in the contemporaneous matched cohort. Together, these findings suggest that the crude between-platform differences in nerve-related complications were largely driven by the early adoption period rather than demonstrating an inherent disadvantage of TOETVA. However, the absence of events after matching also precludes precise between-platform estimation of these rare complications.

The propensity score-matched analysis was particularly informative because it changed the interpretation of the operative-time difference suggested by the crude data. Operative time was shorter in the TORT group in the overall cohort, but this apparent advantage was not maintained after restriction to the contemporaneous period and adjustment for measured confounders. Instead, TORT required approximately 5 min longer than TOETVA after matching. The central compartment node yield remained higher with TORT, but the adjusted difference was less than one node on average. Hospital stay differed by only 0.10 days, a difference that was not statistically significant, and no significant histopathological differences were observed among malignant matched pairs. Thus, although some adjusted differences remained statistically significant, their absolute magnitudes were small and, in isolation, do not establish clinically meaningful superiority of either platform.

Temporal and learning effects are central to interpreting these findings. TOETVA was introduced in September 2016, whereas TORT was introduced in March 2020, after substantial prior transoral experience had already been accumulated. Because the two approaches share vestibular access, flap creation, and a craniocaudal operative view, prior TOETVA experience likely provided procedural familiarity relevant to subsequent TORT adoption. Nevertheless, thyroidectomy during TORT also requires adaptation to robotic instrumentation and platform-specific manipulation (1). Consistent with this distinction, the estimated between-platform difference in operative time remained similar after additional adjustment for calendar time or cumulative transoral experience, whereas exploratory analyses within the TORT cohort demonstrated nonlinear changes in total operative, docking, and console times with increasing robot-specific experience. These findings suggest continued adaptation after robotic adoption without supporting a discrete proficiency threshold.

The results favor viewing TOETVA and TORT as complementary rather than one approach superseding the other. TOETVA does not require access to a robotic platform and may therefore remain particularly relevant where robotic resources or cost considerations limit TORT use. TORT may likewise remain appropriate when robotic resources are available, guided by surgeon experience, patient preference, and case-specific considerations. However, because costs were not systematically collected, the present data do not permit formal comparison of costs or cost-effectiveness between the two platforms.

This study has some limitations. First, its retrospective design and nonrandom platform allocation remain susceptible to residual and unmeasured confounding despite restriction to a contemporaneous cohort and propensity score matching; matching can address only measured covariates. Second, no conventional open thyroidectomy comparator was included, and the present findings therefore should not be interpreted as demonstrating equivalence or superiority of transoral thyroidectomy relative to open surgery. Third, the cohort was highly selected and overwhelmingly comprised patients with pathological stage I disease, limiting generalizability to patients with locally advanced thyroid cancer. Fourth, complication events were uncommon, resulting in limited precision for comparisons of rare adverse outcomes. Fifth, long-term oncological outcomes, including recurrence and disease-free survival, were not systematically evaluated across the entire cohort, precluding robust assessment of these outcomes. Sixth, formal cost and cost-effectiveness analyses were not performed. Seventh, although the single-surgeon setting improves procedural consistency, it may limit generalizability to centers with different levels of transoral or robotic experience. Finally, RLN dysfunction was evaluated by laryngoscopy when clinically indicated rather than routinely in all patients, and mental nerve dysfunction was assessed clinically without formal objective sensory testing.

Longer-term studies incorporating oncological outcomes, standardized patient-reported measures, and formal economic evaluation are warranted to further define the respective roles of TOETVA and TORT, particularly with respect to scar concealment, cosmetic satisfaction, and thyroid-specific quality of life (10, 16–19).

In conclusion, transoral thyroidectomy in this large cancer-enriched single-surgeon cohort was associated with acceptable perioperative outcomes in carefully selected patients. Although TOETVA and TORT showed differences in the overall cohort, adjustment for case mix and operative era yielded only modest between-platform differences and did not establish superiority of either technique. Both approaches may retain practical value within an evolving transoral thyroidectomy practice, with platform selection individualized according to clinical context, surgeon experience, patient preference, and resource availability.

## Data Availability

The datasets used and/or analyzed during the current study are available from the corresponding author on reasonable request.
