# Supplementary material for: Transoral thyroidectomy in a consecutive cancer-enriched cohort of 1–000 patients: a single-surgeon experience with endoscopic and robotic platforms
Source: Front Endocrinol (Lausanne). 2026 Sep 30;17:1877246. doi: 10.3389/fendo.2026.1877246 (PMC13661338; doi:10.3389/fendo.2026.1877246)
Supplement: Supplementary file 1 [file Supplementaryfile1.docx]

**Supplementary Figure Legends**

**Supplementary Figure S1. Covariate balance before and after propensity score matching.** Love plot showing absolute standardized mean differences for the covariates used in propensity score matching before and after matching in the contemporaneous cohort. The dashed vertical line indicates an absolute standardized mean difference of 0.10, the threshold for acceptable covariate balance. Extent of surgery was matched exactly.

Abbreviations: ASMD, absolute standardized mean difference; BMI, body mass index; PSM, propensity score matching; TORT, transoral robotic thyroidectomy.

**Alt text:** Love plot comparing absolute standardized mean differences before and after propensity score matching for sex, age, BMI, nodule size, thyroiditis, extent of surgery, and months since TORT introduction. All covariates had absolute standardized mean differences below 0.10 after matching.

**Supplementary Figure S2. Associations between cumulative robotic experience and operative times during transoral robotic thyroidectomy.** Restricted cubic spline analyses showing the associations between the number of prior TORT cases and (A) total operative time, (B) docking time, and (C) console time. Gray points represent individual observations, solid lines represent model-predicted values, and shaded areas indicate 95% confidence intervals. P values for the overall association and nonlinearity are shown in each panel.

Abbreviation: TORT, transoral robotic thyroidectomy.

**Alt text:** Three-panel figure showing restricted cubic spline analyses of operative times according to cumulative TORT experience. Panel (A) shows total operative time, panel (B) docking time, and panel (C) console time according to the number of prior TORT cases. All three operative-time measures showed significant nonlinear associations with cumulative robotic experience.
