# Supplementary material for: Transoral thyroidectomy in a consecutive cancer-enriched cohort of 1–000 patients: a single-surgeon experience with endoscopic and robotic platforms
Source: Front Endocrinol (Lausanne). 2026 Sep 30;17:1877246. doi: 10.3389/fendo.2026.1877246 (PMC13661338; doi:10.3389/fendo.2026.1877246)
Supplement: Supplementary file 2 [file Supplementaryfile2.docx]

**Supplementary Table S1.** Covariate balance before and after propensity score matching

| **Variable** | **Before matching** | | | **After matching** | | |
| --- | --- | --- | --- | --- | --- | --- |
|  | **TOETVA**  **(n = 267)** | **TORT**  **(n = 550)** | **ASMD** | **TOETVA**  **(n = 221)** | **TORT**  **(n = 221)** | **ASMD** |
| Sex, female, n (%) | 245 (91.8) | 496 (90.2) | 0.055 | 200 (90.5) | 198 (89.6) | 0.030 |
| Age, years | 37.16 ± 8.22 | 37.45 ± 8.29 | 0.035 | 37.22 ± 8.04 | 37.41 ± 8.26 | 0.023 |
| BMI, kg/m² | 22.84 ± 3.51 | 23.03 ± 3.83 | 0.050 | 23.03 ± 3.57 | 22.68 ± 3.48 | 0.099 |
| Nodule size, cm | 0.74 ± 0.41 | 0.88 ± 0.51 | 0.303 | 0.77 ± 0.43 | 0.81 ± 0.46 | 0.090 |
| Thyroiditis, n (%) | 89 (33.3) | 188 (34.2) | 0.018 | 72 (32.6) | 70 (31.7) | 0.019 |
| **Extent of surgery, n (%)** |  |  |  |  |  |  |
| Total thyroidectomy with CCND | 16 (6.0) | 42 (7.6) | 0.065 | 13 (5.9) | 13 (5.9) | 0.000 |
| Lobectomy with CCND | 223 (83.5) | 449 (81.6) | 0.050 | 187 (84.6) | 187 (84.6) | 0.000 |
| Isthmusectomy with CCND | 28 (10.5) | 55 (10.0) | 0.016 | 21 (9.5) | 21 (9.5) | 0.000 |
| Months since TORT introduction | 24.92 ± 14.12 | 33.62 ± 11.00 | 0.687 | 28.59 ± 12.51 | 28.99 ± 12.22 | 0.032 |

The unmatched comparison was restricted to the contemporaneous cohort beginning with the introduction of TORT; 267 TOETVA and 550 TORT procedures were eligible. Propensity scores were estimated using sex, age, BMI, nodule size, thyroiditis, and months since TORT introduction; extent of surgery was matched exactly. One-to-one nearest-neighbor matching without replacement yielded 221 matched pairs. Four TORT cases involving nodulectomy or lobectomy plus nodulectomy had no corresponding TOETVA procedure category and were therefore not retained after matching. An ASMD <0.10 was considered acceptable balance.

Abbreviations: BMI, body mass index; CCND, central compartment neck dissection; ASMD, absolute standardized mean difference; TORT, transoral robotic thyroidectomy; TOETVA, transoral endoscopic thyroidectomy via the vestibular approach.

**Supplementary Table S2.** Histopathological characteristics of malignant cases in the propensity score-matched cohort

| **Variable** | **TOETVA (n = 212)** | **TORT (n = 212)** | **Difference, TORT – TOETVA (95% CI)** | **p-value** |
| --- | --- | --- | --- | --- |
| Gross extrathyroidal extension, n (%) | 2 (0.9) | 1 (0.5) | −0.5 (−2.1 to 1.1) | 0.563 |
| Lymphatic invasion, n (%) | 54 (25.5) | 58 (27.4) | 1.9 (−5.7 to 9.5) | 0.627 |
| Vascular invasion, n (%) | 43 (20.3) | 41 (19.3) | −0.9 (−7.5 to 5.6) | 0.777 |
| T category, n (%) |  |  |  |  |
| T1a | 174 (82.1) | 167 (78.8) | −3.3 (−10.3 to 3.7) | 0.353 |
| T1b | 33 (15.6) | 40 (18.9) | 3.3 (−3.8 to 10.4) | 0.361 |
| T2 | 3 (1.4) | 4 (1.9) | 0.5 (−1.6 to 2.5) | 0.655 |
| T3b | 2 (0.9) | 1 (0.5) | −0.5 (−2.1 to 1.1) | 0.563 |
| N1a, n (%) | 59 (27.8) | 56 (26.4) | −1.4 (−9.6 to 6.8) | 0.736 |
| Stage I, n (%) | 212 (100.0) | 212 (100.0) | 0.0 | — |
| BRAF mutation positive, n/N (%) | 179/210 (85.2) | 167/210 (79.5) | −5.7 (−13.1 to 1.7) | 0.132 |

Histopathological comparisons were restricted to 212 matched pairs in which both patients had malignant disease. BRAF mutation status was compared in 210 matched pairs with available test results in both patients. Differences for categorical variables are absolute percentage-point differences calculated as TORT minus TOETVA. Risk differences and P values were estimated using generalized estimating equations with robust standard errors to account for the matched-pair structure.

Retrieved central compartment node yield is reported in Table 4 and is not duplicated here.

Abbreviations: CI, confidence interval; TOETVA, transoral endoscopic thyroidectomy via the vestibular approach; TORT, transoral robotic thyroidectomy.

**Supplementary Table S3.** Sensitivity analyses for total operative time in the propensity score-matched cohort

| **Additional adjustment** | **TOETVA adjusted mean ± SE, min** | **TORT adjusted mean ± SE, min** | **Mean difference, TORT – TOETVA (95% CI)** | **p- value** |
| --- | --- | --- | --- | --- |
| None beyond PSM | 65.26 ± 1.67 | 70.31 ± 1.77 | 5.05 (0.39 to 9.72) | 0.034 |
| Continuous calendar time | 65.14 ± 1.68 | 70.43 ± 1.62 | 5.29 (0.62 to 9.95) | 0.026 |
| Cumulative transoral experience | 65.21 ± 1.67 | 70.36 ± 1.66 | 5.14 (0.47 to 9.82) | 0.031 |

Calendar time was defined as months since TORT introduction. Cumulative transoral experience was defined as the number of prior TOETVA and TORT procedures. Calendar time and cumulative transoral experience were highly correlated (Pearson r = 0.98, P < 0.0001), with substantial multicollinearity when included simultaneously (variance inflation factor approximately 24). They were therefore examined in separate models. All models used generalized estimating equations with robust standard errors to account for the matched-pair structure. Positive mean differences indicate longer operative time in TORT than TOETVA.

Abbreviations: CI, confidence interval; SE, standard error; TOETVA, transoral endoscopic thyroidectomy via the vestibular approach; TORT, transoral robotic thyroidectomy.
